# Investment preference promotes cooperation in spatial public goods game

**DOI:** 10.1371/journal.pone.0206486

**Published:** 2018-11-14

**Authors:** Shuhua Chang, Zhipeng Zhang, Yu Li, Yu E. Wu, Yunya Xie

**Affiliations:** Coordinated Innovation Center for Computable Modeling in Management Science, Tianjin University of Finance and Economics, Tianjin, China; University of Zaragoza, SPAIN

## Abstract

It is usually assumed that each cooperator contributes equally to different public pools in spatial public goods game. However, it is more reasonable to invest differently according to individual investment preference. In this paper, an extended public goods game, in which cooperators contribute to the groups according to the investment preference, is developed. The investment preference of a cooperator is characterized by the fraction of the cooperator from his/her own memory about a group and the intensity of investment preference is represented by a tunable parameter *α*. The well-mixed population and the structured population are analyzed under this mechanism. It is shown that the investment preference can give rise to coordination. Moreover, the extensive numerical simulation results show that with the increasing of investment preference density or memory length, the proportion of cooperation can increase monotonously. This is because the investment preference could help cooperators resist the invasion from defectors. Compared with the basic version, the new mechanism is able to promote cooperation effectively. Our research may provide a valuable insight for further exploring the nature of cooperation in the real world.

## Introduction

The evolution of cooperative behavior is one of the most important problems in the field of social and biological sciences [1]. Evolutionary game theory [2, 3] is one of the most fruitful frameworks to investigate the emergence and sustainability of cooperation. Particularly, interactions of two-person dilemmas are usually modeled with the prisoner’s dilemma game (PDG) and the snowdrift game (SDG) [4–8]. For multi-individual interactions, a primary research model is the so called public goods game (PGG) [9–12]. In a PGG, players are likely to make a contribution to the public pool. The total contributions in the public pool will be multiplied by the synergy factor *r* (1 < *r* < *N*). After that the product is equally distributed to all participants irrespective of their strategies. Apparently, the free riders (the players who do not invest) will dominate the whole group [13–15] and evolutionary game theory predicts that the destruction of cooperation will lead to “tragedy of the commons” [16].

Based on the evolutionary game theory, many factors and mechanisms have been proposed to explain the emergence and sustainability of cooperation, such as, social diversity [17–20], punishment [21–26], voluntary participation [27, 28], conditional strategies [29], reputation [30–32], reward [9] and so on [33–37]. An outstanding work by Nowak and May [38] shows that the simple two-dimensional spatial structure is able to maintain cooperation in the PDG. Following this fruitful line, much attention has been paid to the PGG including the square lattice [39–41], small-world networks [42, 43], scale-free networks [44–46], and adaptive networks [47–50]. Nowak attributes all these to the following mechanisms: kin selection, direct reciprocity, indirect reciprocity, network reciprocity, and group selection [51].

In the previous works [52–54], the contribution of a cooperator is the same for each group. However, an investor will have a preference for some industries in the real world, instead of investing in different industries equally. Experiments show that cooperative behavior is enhanced as a result of social preferences guided by simple decision heuristics [55]. So, it is necessary to further explore the investment process. Furthermore, the memory [56] about historical information is also an important factor for investment. For example, one investor may invest more capitals in the high yielding places according to his/her past memories. Therefore, it is reasonable to relax this assumption.

In the present work, the important objectives are the introduction of the memory length about the historical information and the investment preference of agents. As a consequence, we propose a new evolutionary PGG model based on the above considerations. That is to say, the value of individual investment is proportional to the fraction of the cooperator in the group. History information (the fraction of cooperators) from the repeated interactions is decided by the length of individual memory. Tit-for-tat and win-stay, lose-shift are two classic strategies depending on the previous memory of players [57]. Direct reciprocity is an non-negligible mechanism in the spatial evolutionary game [58]. Moreover, memory capacity can be combined with a lot of factors, for example, intention recognition [59–62], which makes it possible for intention recognizers to predict the next movement of the opponents based on past memory. Similarly, a prior commitment strategy [63–66] is also proposed and high level of cooperation can be achieved. Obviously, it is when players change strategies that the above papers will consider memory. However, this new mechanism does not solely benefit from direct reciprocity. Differently, here we consider that all individuals have memory capacity and they can use this capacity when they make a rational investment. For example, even if a player is a defector in the current round, he/she still knows the proportion of cooperators within the group, and if he/she will choose to be a cooperator himself/herself in the next round, he/she can also use this information about the proportion of cooperators to make investment decisions. The investment model returns to the traditional case if all players are cooperators in a group. However, with the new model, the fraction of cooperation reaches a level which can not be found in the traditional version. Cooperators give a signal to defectors by changing the investment. Finally, an adjustable control parameter is used to represent the extent of individuals’ preference to various groups.

More importantly, this idea is more reasonable compared with unified preference pattern. As we will show, this investment preference leads to coordination so that cooperators are able to restrict defectors’ invasion. To understand this mechanism clearly, we first study it by means of the replicator equation in well mixed populations. Next, the results of Monte Carlo simulations in structured populations are also analyzed. We show the public goods game with investment preference in the Methods before we present the results in detail.

## Methods

In the present paper, we allow a cooperator to adjust the value of his/her contribution *c*_*i*_(*t*) dynamically in the public goods game. In particular, if player *i* is a cooperator, he/she will contribute *c*_*i*_(*t*) to the common pool at step *t*, and *c*_*i*_(*t*) depends on the fraction of cooperators in the group which he/she belongs to and his/her memory length. What is more, we also observe how the investment preference density influences the evolution of cooperation. Therefore, the cost of cooperator *i* at each step *t* is given by

$$\begin{array}{c} c_{i}\left(t\right)={\left(\frac{\sum_{p=1}^{M}c*n_{ic}\left(t-p\right)}{M(k_{i}+1)}\right)}^{\alpha }, \end{array}$$ (1)

where *M* (*M* = 1, 2, 3, …) is the memory length, *p* (*p* = 1, …, *M*) is the backtracking time, *n*_*ic*_ is the number of cooperators in the group that player *i* participates, *c* = 1 is the fixed cost, *k*_*i*_ is the number of neighbors (degree), and *α* (*α* ≤ 1) is a tunable density parameter. Evidently, Eq (1) illustrates that investment preference is invalid for *α* = 0. For *α* > 0, however, a better investment mode is allowed for cooperators.

### Well-mixed populations

Suppose that there are a fraction of cooperators *f*_*c*_ and 1 − *f*_*c*_ defectors in infinitely large well-mixed populations. According to the replicator dynamics [67, 68], the evolution of *f*_*c*_ is determined by Eq (2)

$$\begin{array}{c} \dot{f_{c}}=f_{c}\cdot \left(1-f_{c}\right)\cdot \left[P_{C}-P_{D}\right], \end{array}$$ (2)

where $\dot{f_{c}}$ is the gradient of selection [69] for cooperation, *P*_*C*_ and *P*_*D*_ are the average payoff of cooperators and defectors. An interaction of the game consists of *n* players chosen at random according to binomial sampling from the populations. As a consequence, *P*_*C*_ and *P*_*D*_ are determined by the following equations, respectively:

$$\begin{array}{c} P_{C}=\sum_{n_{c}=0}^{n-1}C_{n-1}^{n_{c}}f_{c}^{n_{c}}{\left(1-f_{c}\right)}^{n-1-n_{c}}(\frac{c_{i}\cdot \left(n_{c}+1\right)\cdot r}{n}-c_{i}), \end{array}$$ (3)

$$\begin{array}{c} P_{D}=\sum_{n_{c}=0}^{n-1}C_{n-1}^{n_{c}}f_{c}^{n_{c}}{\left(1-f_{c}\right)}^{n-1-n_{c}}\frac{c_{i}\cdot n_{c}\cdot r}{n}, \end{array}$$ (4)

where *n*_*c*_ (*n*_*c*_ = 0, …, *n* − 1) is the number of cooperators among the *n* − 1 other players in a group, $C_{n-1}^{n_{c}}f_{c}^{n_{c}}$ is the probability with which there are *n*_*c*_ cooperators, and *r* is the enhancement factor. In addition, the terms $\left(\frac{c_{i}\cdot \left(n_{c}+1\right)\cdot r}{n}-\frac{n_{c}+1}{n}\right)$ in Eq (3) represent the payoff of a cooperator, and the term $\frac{c_{i}\cdot n_{c}\cdot r}{n}$ in Eq (4) is the payoff of a defector in a group that contains *n*_*c*_ cooperators.

### Structured populations

Here we also consider the evolutionary PGG on a square lattice with periodic boundary conditions and of size *L* × *L*. Initially, each node is designed as either a cooperator or a defector with equal probability. Every player will take part in the (*k*_*i*_+ 1) PGGs centered on his four neighbors and centered on himself, where *k*_*i*_ = 4 is the degree of player *i* on the regular network.

Subsequently, the sum of contributions accumulated within one group is multiplied by the factor *r* and the product is redistributed to the 5 players equally. Correspondingly, an individual could gain a payoff as follows:

$$\begin{array}{c} P_{i}^{g}\left(t\right)=\{\begin{array}{cc} \frac{r\cdot \sum_{i\in g}S_{i}\cdot c_{i}\left(t\right)}{k_{i}+1}-S_{i}\cdot c_{i}\left(t\right), & \text{if}\;S_{i}=C,\\ \frac{r\cdot \sum_{i\in g}S_{i}\cdot c_{i}\left(t\right)}{k_{i}+1}, & \text{if}\;S_{i}=D, \end{array} \end{array}$$ (5)

where *S*_*i*_ is player *i*’s strategy and $P_{i}^{g}\left(t\right)$ is *i*’s payoff getting from group *g* at time *t* according to Eq (5). Here *c*_*i*_(*t*) is changing constantly according to Eq (1). Each selected player *i* could acquire the payoff *P*_*i*_(*t*) at time *t* from the five groups (*G* = 5) with which it is affiliated and the total payoff is expressed by Eq (6):

$$\begin{array}{c} P_{i}\left(t\right)=\sum_{g=1}^{G}P_{i}^{g}\left(t\right). \end{array}$$ (6)

We can easily find some examples to explain the model. For example, in the human society, an investor will put her/his main funds into the good industries, and stay away from the risky areas. If he thinks that the environment is not conducive to investment, then he will reduce the investment. Meanwhile, the historical information about investment environment influences personal preferences, so we consider the change in the memory length.

Following the previous works [70, 71], at the end of each step player *i* will randomly choose a neighbor *j* as the reference to update its strategy according to the following probability:

$$\begin{array}{c} P=\frac{1}{1+\exp[\left(P_{i}\left(t\right)-P_{j}\left(t\right)\right)/K]}, \end{array}$$ (7)

where *K* represents the intensity of selection, and in this article *K* = 0.5.

The simulation results presented in the Results comprises 100 × 100 individuals. Moreover, in order to exclude the impact of initialization, the fraction of cooperators is determined by averaging over the last 1000 full Monte Carlo steps (MCS) of total 31000 steps. In addition, the final results are averaged by 10 independent runs.

In addition, the simulation is also carried out on BA scale-free networks with network size *N* = 2500 and *m* = *m*_0_ = 2. The equilibrium frequencies of cooperators are obtained by averaging over the last 5000 MCS of total 30000 steps. Each piece of data is averaged over 100 runs.

## Results

**Fig 1:**
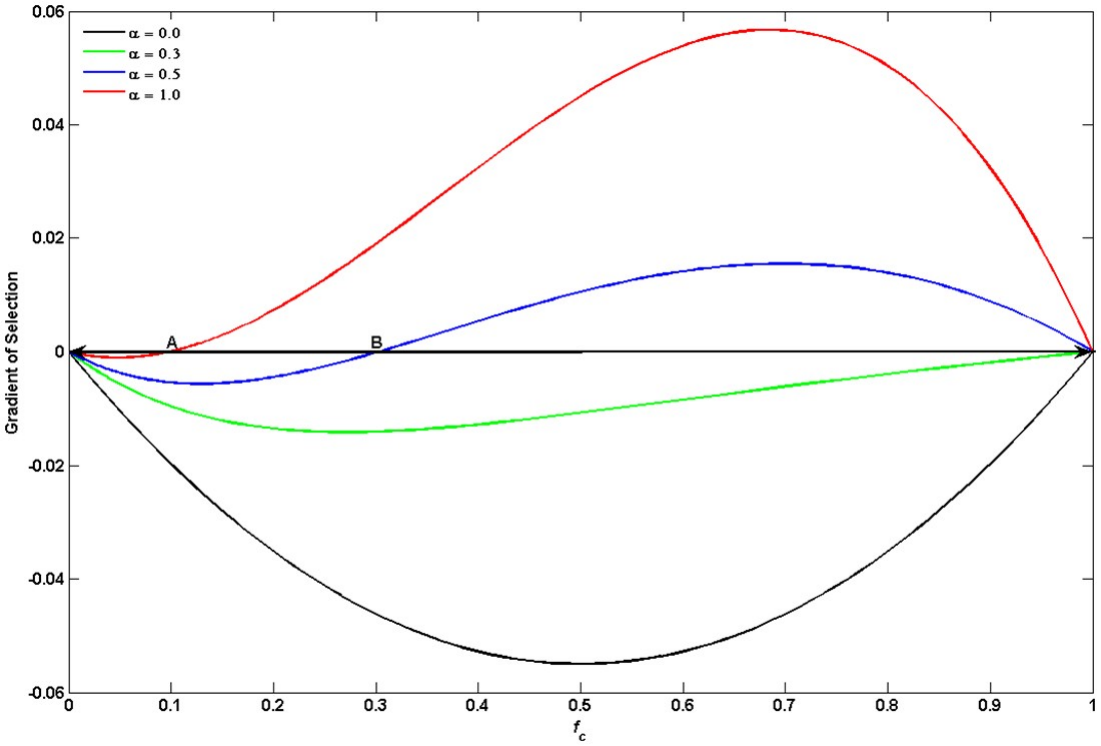
Gradient of selection for the public goods game in well-mixed populations. It shows the results obtained under different values of *α*. With the increasing of *α*, the evolution of cooperation is improved effectively. Parameters are *r* = 3.9, *M* = 1 and *n* = 5.

To better understand the mechanism, we first show the results obtained in well-mixed populations in Fig 1. The gradient of selection ${\dot{f}}_{c}$ is defined in Eq (2), which indicates the evolution of the fraction of cooperators. Here we consider the situation of *M* being equal to 1. We replace the fraction of cooperators in the last round with an average fraction $\frac{n_{c}+1}{n}$ or $\frac{n_{c}}{n}$ because the history information cannot be expressed quantitatively in well-mixed populations. Therefore, Eqs (3) and (4) can be transformed into (8) and (9), respectively. The meaning of the symbols in Eqs (8) and (9) is same as that in Eqs (3) and (4). As shown in Fig 1, the gradient of selection is always negative in the tradition case (*α* = 0). This implies that all players will choose defection gradually and defectors dominate the whole populations finally irrespective of initial conditions. However, the evolution becomes different obviously when investment preference is introduced. For example, although the gradient of selection is negative when *α* is equal to 0.3, the speed of defectors’ expansion turns slow. With the increasing of *α*, it is observed that an unstable point (e.g., A or B) appears, which means that a coordination threshold determines the global dynamics. Under this circumstance, both *f*_*c*_ = 0 and *f*_*c*_ = 1.0 are stable states. The whole populations evolve towards to full cooperation if the initial condition falls on the right side of A (B). On the contrary, full defection is inevitable. This mechanism can facilitate coordination [72] as *α* increases. In addition, it is important to note that *f*_*c*_ = 0 still occupies 10% of space even *α* = 1 for *r* < *n*. According to these results, it could see that this mechanism can promote cooperation in well-mixed populations.

$$\begin{array}{c} P_{C}={\sum_{n_{c}=0}^{n-1}C_{n-1}^{n_{c}}f_{c}^{n_{c}}\left(1-f_{c}\right)}^{n-1-n_{c}}\left(\frac{{\left(\frac{n_{c}+1}{n}\right)}^{\alpha }\cdot \left(n_{c}+1\right)\cdot r}{n}-{\left(\frac{n_{c}+1}{n}\right)}^{\alpha }\right), \end{array}$$ (8)

$$\begin{array}{c} P_{D}=\sum_{n_{c}=0}^{n-1}C_{n-1}^{n_{c}}f_{c}^{n_{c}}{\left(1-f_{c}\right)}^{n-1-n_{c}}\frac{{\left(\frac{n_{c}}{n}\right)}^{\alpha }\cdot n_{c}\cdot r}{n}, \end{array}$$ (9)

**Fig 2:**
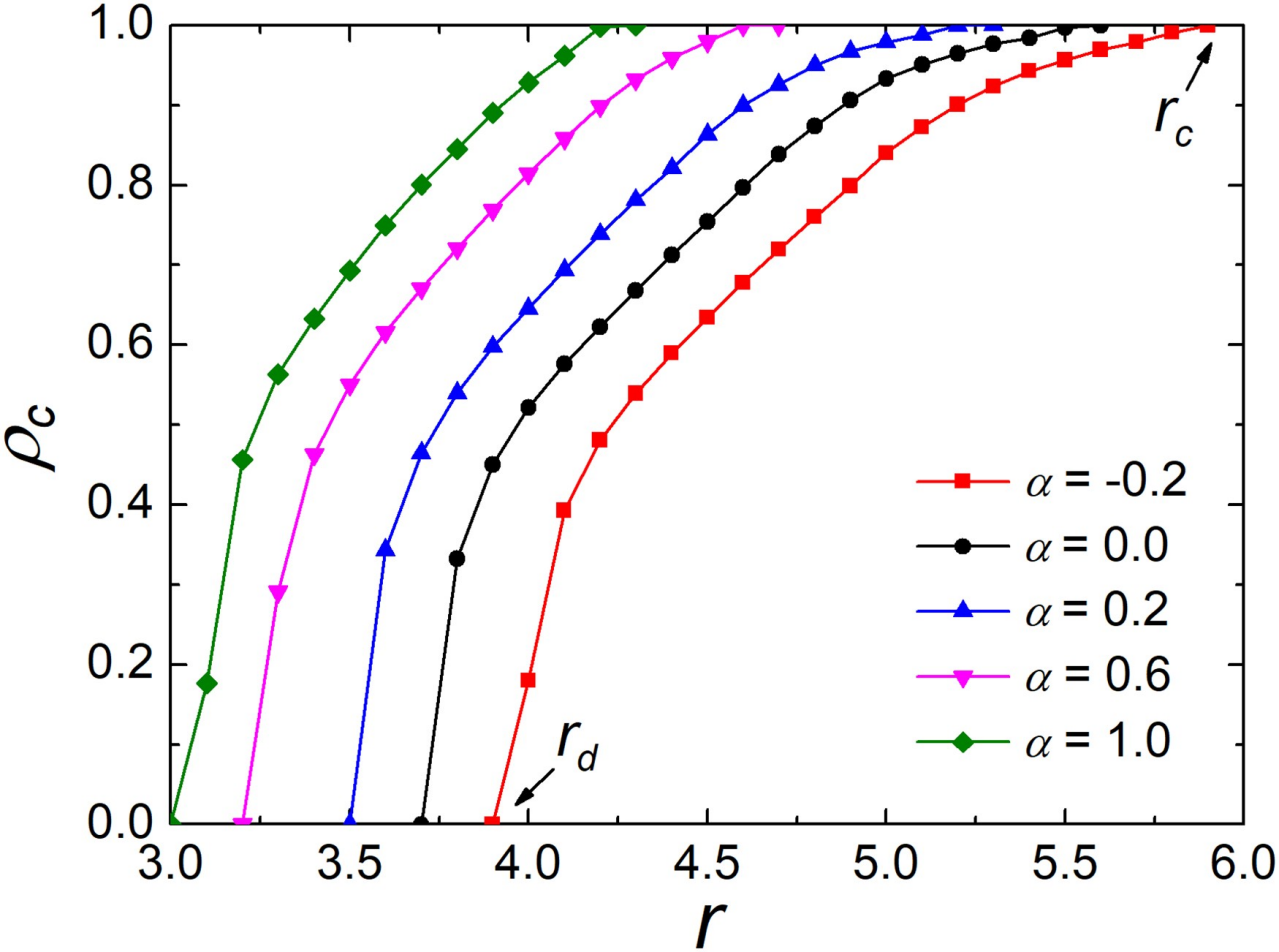
Frequency of cooperators *ρ*_*c*_ depends on *r* for the different values of preference level *α*. The depicted results are obtained for *M* = 1 and *K* = 0.5. The curves show that the proportion of cooperation increases monotonically with *α*.

Next, our focuses are going from well-mixed populations to structured populations. We start by studying the influence of preference level *α* on the evolution of cooperation on a square lattice. Fig 2 shows how the fraction of cooperators *ρ*_*c*_ varies with the enhancement factor *r* for different values of *α*. It is clear that all curves monotonically increase with *r*. This is also obtained in an experimental study [55]. Compared with the traditional case [73] (*α* = 0), the negative *α* undermines the environment of cooperation, while the positive *α*, on the contrary, increases the level of cooperation. In addition, there are two key thresholds *r*_*d*_ and *r*_*c*_, which denote the emergence and full dominance of cooperation, respectively. Namely, if *r* is greater than *r*_*c*_, cooperation will dominate the whole network. On the contrary, cooperation will vanish if *r* is less than *r*_*d*_. For positive *α*, the two key values will move left, while move right for negative *α*. For example, the value of *r*_*c*_ when *α* = 1 is closed to the value of *r*_*d*_ when *α* = −0.2. From Fig 2, cooperators can survive at the condition where cooperators die out in the traditional version. Therefore, these results indicate that investment preference could effectively enhance cooperation in the PGG.

**Fig 3:**
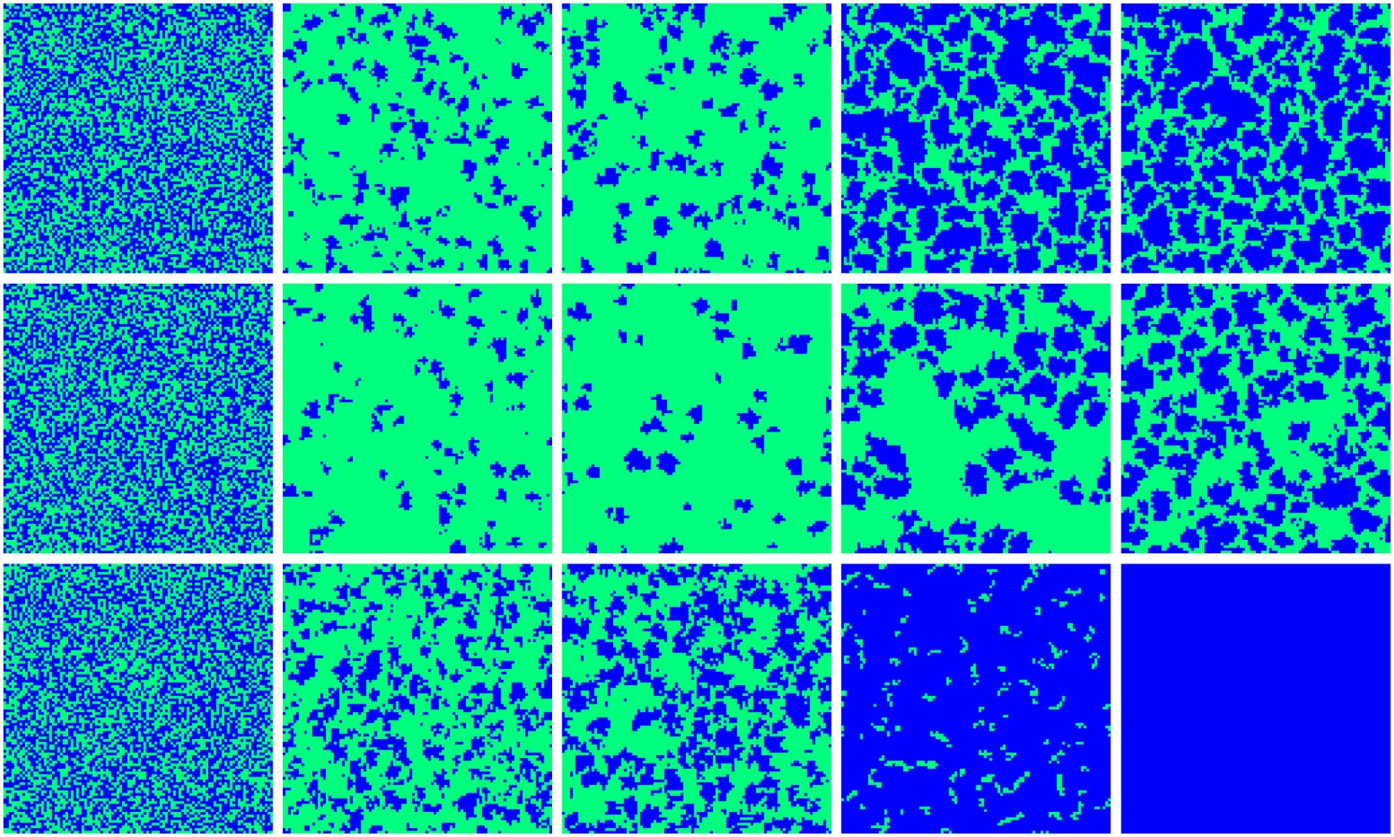
Typical strategy distribution of cooperators (blue) and defectors (green). The snapshots are given for *t* = 0, 5, 10, 100, 10000. The top panels are the traditional version. The middle panels and the bottom panels are the situations for *α* = −0.2 and *α* = 1. The depicted results are obtained for *M* = 1, *K* = 0.5 and *r* = 4.2.

Except for the above expression, it is necessary to show the role of new mechanism via typical snapshots. As shown in Fig 3, three typical values of *α* are listed: *α* = 0 (the first row), *α* = −0.2 (the second row), *α* = 1 (the third row). Initially, cooperators and defectors are evenly distributed on the networks, and there is no any incidence of cooperation clusters. Soon, the number of cooperators sharply decreases and cooperators form small clusters, regardless of *α* values. As the previous literature [74], clusters could protect cooperators against exploitation from defectors. For the traditional case (*α* = 0), the number of cooperation clusters is larger than the case of *α* = −0.2, yet less than the case of *α* = 1.0, which means that the preference level plays a key role in maintaining the number of cooperation clusters at the end of exploitation. However, the following trend becomes greatly different. For the traditional case, the small clusters will expand quickly, take back the lost ground and occupy the most sites of networks. Interestingly, the expanding speed becomes slow for the negative *α*, which not only decreases the number of clusters, but also makes the related sizes of clusters smaller. When the positive *α* is considered, the cooperation clusters will expand faster and form a giant cluster. This giant cluster makes cooperation reach full dominance finally. This is because cooperators can make full use of investment and gain satisfying payoff. As a consequence, the larger the value of *α* is, the more compact the existing clusters are. Based on these findings, it is clear that the preference level decides the cooperation level via the formation and expansion of cooperative clusters.

**Fig 4:**
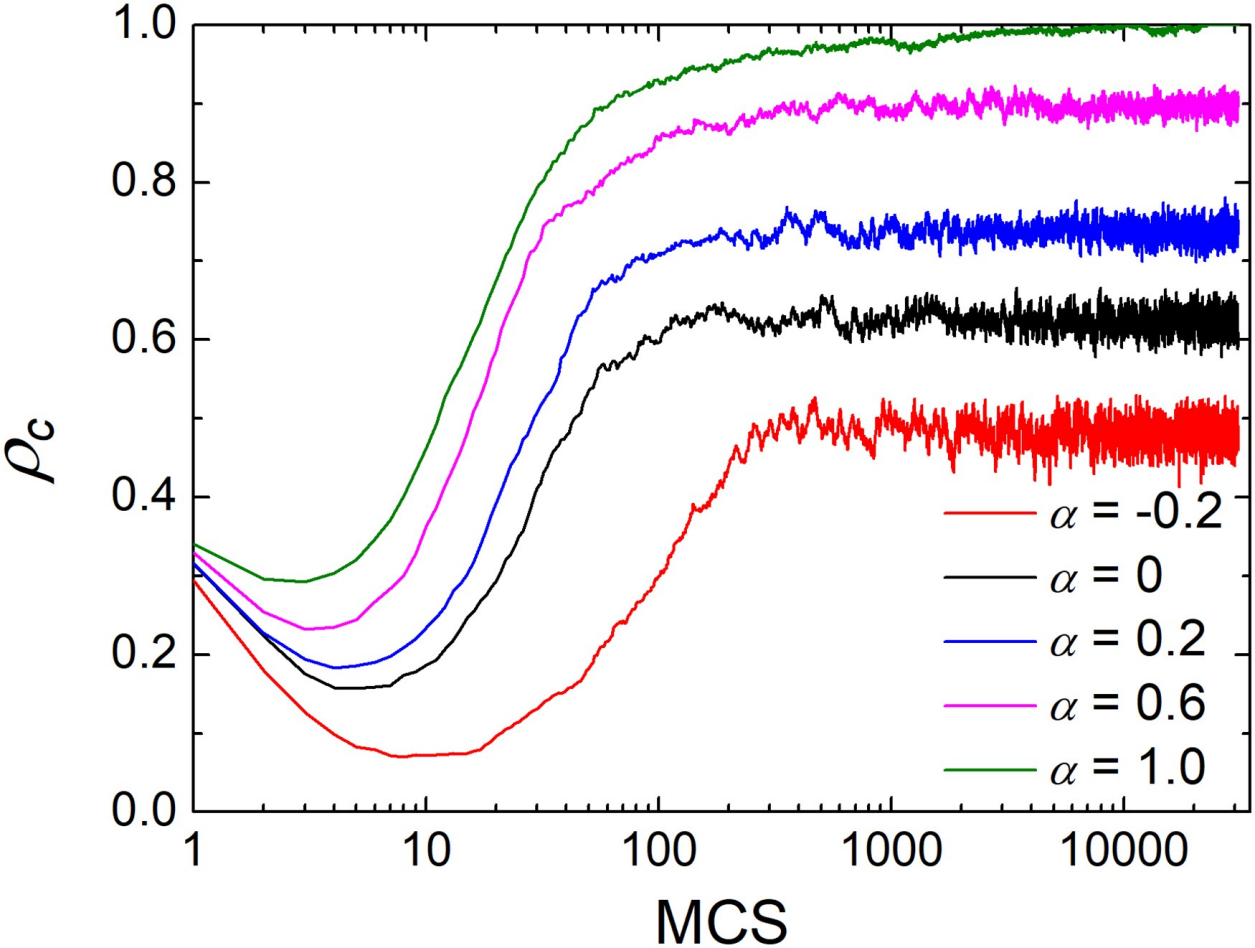
Time series of cooperation for the different values of *α*. All the results are obtained for the enhancement factor *r* = 4.2 and the memory length *M* = 1. The size of square lattice is 100 × 100.

To get quantitative validation for the above visual observations, it is next of interest to explore how cooperation varies with time in Fig 4. Irrespective of which case, cooperation will firstly be exploited by defectors, and the fraction of cooperation declines quickly. This is easy to understand because the benefit of defectors is higher (i.e. defectors could exploit cooperators on account of random distribution of the initially designed strategy). However, the following trend will greatly change. The remaining cooperators will form compact clusters to resist the further invasion and importantly take back the lost territory. As shown in Fig 4, the curves pull up fast and reaches the equilibrium level gradually. Compared with the traditional case (*α* = 0), the positive *α* enables the feedback mechanism more robust and stronger. At the early time, cooperators will be exploited less and form larger clusters (as shown in Fig 3) to reach a higher level of cooperation. While for the negative *α*, the early decline of cooperation will be more obvious and natural, and the later expansion will be more gentle (i.e. lower cooperation). Consequently, together with Fig 3, we can understand the role of preference level: the positive *α* helps cooperation form more compact and larger clusters at the end of exploitation, and they enable the remaining clusters to expand more effectively toward dominance.

**Fig 5:**
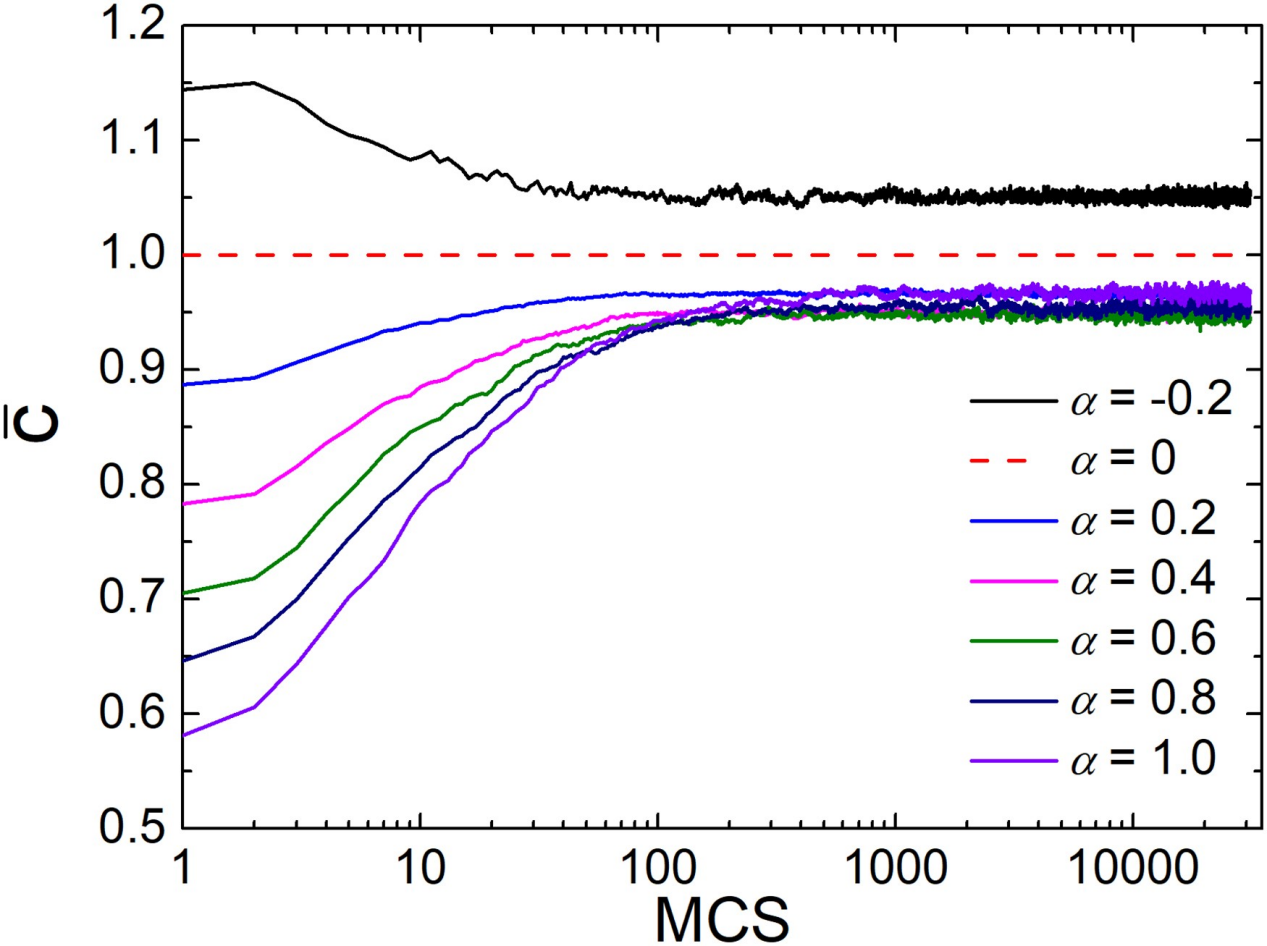
Average investment of cooperators for the different values of *α* as *r* = 4 and *M* = 1. Investment preference changes the investment behaviors effectively. At the same time, investment will keep fluctuation when games reach the equilibrium.

Because this new mechanism is about the investment preference, it is interesting to explore how the preference level *α* affects the average investment. Fig 5 depicts the evolution of average investment for the different values of *α*. Compared with the homogeneous investment of traditional case (the dash line), the negative *α* enhances the average investment (i.e. larger than 1), which can also be derived from Eq (1). At the early stage, players will invest more because cooperators and defectors are fully mixed. However, with the fast formation of clusters, the investment enthusiasm of players also decreases. While for the positive *α*, the average investment shown by five curves in Fig 5 is lower than 1, and the average investment gradually increases, which is related to the robust investment preference mechanism. Besides, the results suggest that the positive *α* optimizes the average investment, which attracts more cooperators to form larger clusters.

**Fig 6:**
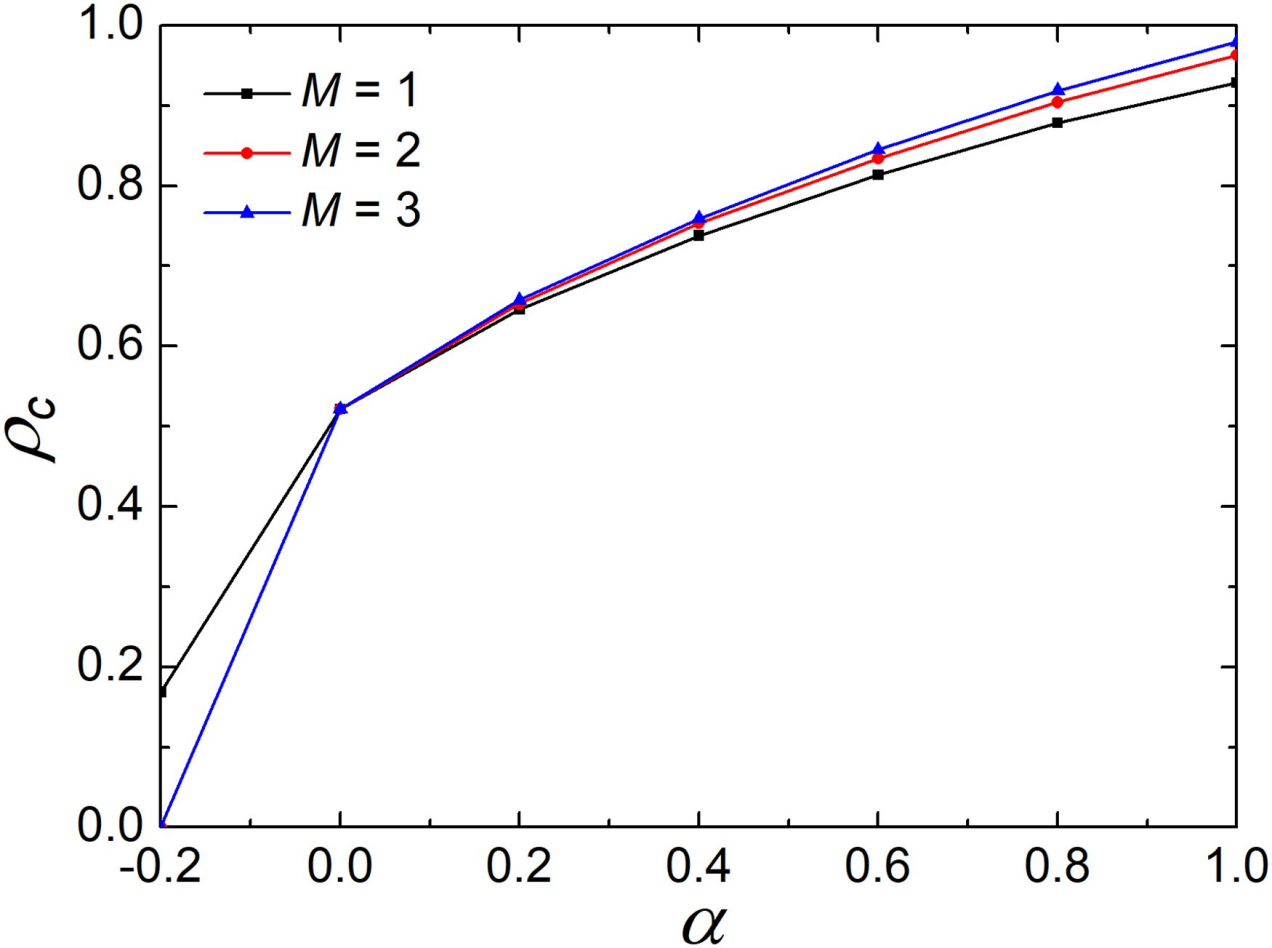
Frequency of cooperators depends on *α* for the different values of *M* when *r* = 4. It is obvious that all the curves monotonically increase, which means that the memory length can promote cooperation but the promotion effect gradually weakens.

Besides the investment preference, what attracts our interest is to check the impact of memory length *M* on the evolution of cooperation. In Fig 6, we depict how cooperation varies as a function of *α* for the different values of *M*. It is clear that the curves of *ρ*_*c*_ are monotonically increasing with the increasing of *α* regardless of *M*. Noteworthily, *α* = 0 is a boundary for the impact of memory length *M*. For the negative *α*, the increase of memory length does not promote cooperation. From Eq (1), it is shown that the larger *M* will generate more investment (>1) under the negative *α*. In this case, the larger investment will lead to the lower frequency of cooperation, which can be easily obtained from Fig 6. However, for the positive *α*, though the difference is slight, the larger *M* will enhance the level of cooperation. Moreover, the gap between *M* = 2 and *M* = 3 is smaller than that between *M* = 1 and *M* = 2. Therefore, it is not hard to imagine that the enhancement effect of memory length will be saturated as *M* increases. Also, based on Eq (1), the larger *M* leads to more appropriate average investment for the positive *α*. Together with Fig 4, it can be seen that investment preference creates a better cooperation environment.

**Fig 7:**
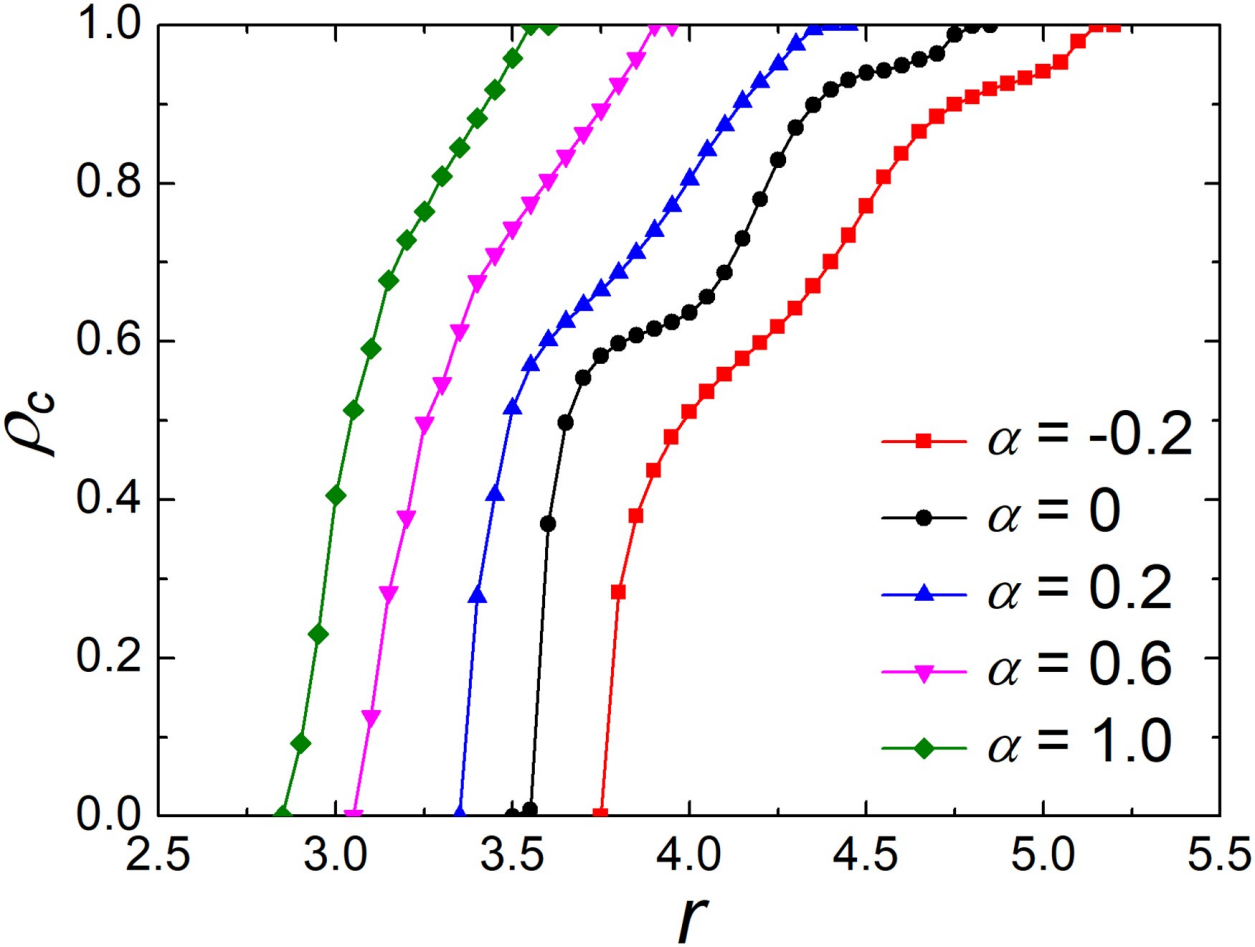
Fraction of cooperators as a function of the investment preference density *α* on BA scale-free networks. Cooperation can still widely spread at a low level of enhanced factor when this new mechanism is introduced. The depicted results are obtained for *M* = 1.

The regular lattice has provided a simplified profile of real interaction networks. However, for example, some individuals can sustain the connections with a lot of players, but the others have few friends in the real world. Social structure exhibits heterogeneity. Consequently, we also simulate this mechanism on BA scale-free networks. As shown in Fig 7, a virtual promotion effect on the evolution of cooperation can be observed compared with the traditional version (*α* = 0). The above results show that this new mechanism could promote cooperation effectively in all studied structures in spatial public goods game.

## Discussion

In the present paper, we have proposed a new model to investigate the impact of preferential investment on the public goods game in well-mixed and structured population. In detail, a cooperator makes a contribution to the public pool according to the number of cooperators in his/her group and the memory length. Through numerous computational simulations, we unveil that the new method could effectively enhance cooperation. In addition, the threshold of cooperation survival, as we can observe, is closely related to the formation of compact cooperation clusters and the investment preference mechanism. For the negative preference level, the clusters at the end of exploitation period are slim, which could not guarantee the strong recovery of cooperation. However, the positive *α* will shorten the exploitation period, which forms more compact clusters. These clusters will expand more robustly and form giant clusters, finally lead to the full dominance of cooperation. In addition, we find that the preferential level also affects the average investment. The negative preference enhances the average investment, which can not inhibit the decline of cooperation. The present results show that despite cooperation being enhanced with the introduction of the proposed mechanism in all studied structures, there are indications that the nature of the population-wide dynamics is sensitive to underlying topology of the network. For example, coordination and co-existence are observed respectively in different topologies [17, 67].

Notably, our new public goods game seems very reasonable because people usually adjust their investment directions according to the past information. In addition, investment preference *α* is likely to change according to different factors. However, we just consider the simplest model. We hope that this research can positively gain new insights to the realistic social dilemma and inspire future studies.
